# Correction: Seek and you may (not) find: A multi-institutional analysis of where research data are shared

**DOI:** 10.1371/journal.pone.0306199

**Published:** 2024-06-21

**Authors:** Lisa R. Johnston, Alicia Hofelich Mohr, Joel Herndon, Shawna Taylor, Jake R. Carlson, Lizhao Ge, Jennifer Moore, Jonathan Petters, Wendy Kozlowski, Cynthia Hudson Vitale

The ORCID iDs are missing for the first, fourth, fifth, sixth, seventh and ninth author. Please see the authors’ respective ORCID iDs here:

- Lisa R. Johnston’s ORCID iD is: 0000-0001-6908-9240
  (https://orcid.org/0000-0001-6908-9240)
- Shawna Taylor’s ORCID iD is: 0000-0002-9842-7867
  (https://orcid.org/0000-0002-9842-7867)
- Jake Carlson’s ORCID iD is: 0000-0003-2733-0969
  (https://orcid.org/0000-0003-2733-0969)
- Lizhao Ge’s ORCID iD is: 0009-0005-7862-6016
  (https://orcid.org/0009-0005-7862-6016)
- Jennifer Moore’s ORCID iD is: 0000-0001-6628-6820
  (https://orcid.org/0000-0001-6628-6820)
- Wendy Kozlowski’s ORCID iD is: 0000-0001-6539-3798
  (https://orcid.org/0000-0001-6539-3798)

Consequently, in the How complete are the metadata records associated with these datasets? subsection of Results, there is an error in the first bullet after second paragraph. The correct first bullet is: Digital Object Identifier (DOI): The persistent unique identifier (PID) for the dataset minted by the repository.
