# Supplementary material for: Seek and you may (not) find: A multi-institutional analysis of where research data are shared
Source: PLoS One. 2024 Apr 25;19(4):e0302426. doi: 10.1371/journal.pone.0302426 (PMC11045069; doi:10.1371/journal.pone.0302426)
Supplement: Attachment — Submitted filename: Response to Reviewers.docx [file pone.0302426.s001.docx]

Dear Reviewers,

Thank you for the feedback and the opportunity to submit this letter in response to the initial comments to our manuscript “Seek and you may (not) find: A multi-institutional analysis of where research data are shared”.

Reviewers provided the comment below, what follows is our response to these comments:

*“While bibliometric data can be used as a basis to evaluate publication activity in a given discipline, we feel that in order to justify consideration as primary research, bibliometric studies should address a clear and meaningful research question, provide an adequate rationale for the study, include a well-reported study design, and contribute to the base of knowledge by presenting data of utility to the scientific community.”*

This article is based on original research conducted as part of a National Science Foundation EAGER study (#2135874) which sought to answer the following questions:

RQ1: Where are research data being shared?

RQ2: How many datasets are being shared?

RQ3: How complete are the metadata records associated with these datasets?

We have added a section to the revised manuscript to articulate our research questions and have added headings in the results section to connect the questions with the related outcomes.

These questions and the resulting data are significant to the science of science research community, international policy makers, institutional stakeholders, and others, given the expected exponential growth of research data management and sharing practices in the United States as a result of the 2022 US Office of Science and Technology Policy (OSTP) memo, [Ensuring Free, Immediate, and Equitable Access to Federally Funded Research](https://www.whitehouse.gov/wp-content/uploads/2022/08/08-2022-OSTP-Public-access-Memo.pdf), and, globally, with the adoption of the 2021 [UNESCO Recommendation on Open Science](https://www.unesco.org/en/open-science/about?hub=686). We have added this additional rationale to the manuscript.

Based on the reviewer feedback we’ve also clarified and summarized our methods section. As detailed in our paper, our overall study design employed the following research methods:

1. Leverage the DataCite and CrossRef API’s to identify institutionally-related datasets and software code;
2. Aggregate, clean, and normalize the resulting metadata to calculate estimates of the number and locations of shared data across institutions;
3. Evaluate the completeness of the DataCite metadata records in describing information essential for federal funded datasets,as recommended in the OSTP memo

Finally, in our introduction and conclusion we have connected the outcomes and utility of this research to the scientific community. In brief, by understanding where researchers share their data and the resulting quality of the metadata, better meta-analyses on the scientific record may be performed and science itself can be accelerated. As described by Fortunato, et.al. ([Nature, 2018](https://www.ncbi.nlm.nih.gov/pmc/articles/PMC5949209/)):

“The science of science (SciSci) offers a quantitative understanding of the interactions among scientific agents across diverse geographic and temporal scales: It provides insights into the conditions underlying creativity and the genesis of scientific discovery, with the ultimate goal of developing tools and policies that have the potential to accelerate science.”

Again, we appreciate the opportunity to respond to reviewers’ initial comments and have revised our manuscript to better reflect our research and their concerns.

Respectfully,

Joel Herndon*, Lisa R. Johnston, Alicia Hofelich Mohr, Jake R. Carlson, Lizhao Ge, Jennifer Moore, Jonathan Petters, Joel Herndon, Wendy Kozlowski, Shawna Taylor, and Cynthia Hudson Vitale

*Corresponding Author: joel.herndon@duke.edu
